# Dopaminergic foundations of schizotypy as measured by the German version of the Oxford-Liverpool Inventory of Feelings and Experiences (O-LIFE)—a suitable endophenotype of schizophrenia

**DOI:** 10.3389/fnhum.2013.00001

**Published:** 2013-01-24

**Authors:** Phillip Grant, Yvonne Kuepper, Eva A. Mueller, Catrin Wielpuetz, Oliver Mason, Juergen Hennig

**Affiliations:** ^1^Personality Psychology and Individual Differences, Department of Psychology, Justus-Liebig-University GiessenGiessen, Germany; ^2^Research Department of Clinical, Educational and Health Psychology, University College LondonLondon, UK

**Keywords:** schizotypy, schizophrenia, endophenotype, O-LIFE, dopamine, genetic associations, psychosis

## Abstract

The concept of schizotypy or “psychosis proneness” captures individual differences in perceptual, cognitive, and affective experiences that may relate to a range of psychotic disorders. The concept is an important way to assess the contribution of pre-existing psychological and genetically based biological features to the development of illnesses such as schizophrenia (so called endophenotypes). The Oxford-Liverpool Inventory of Feelings and Experiences (O-LIFE) is a widely used multi-dimensional measure of the construct and consists of four scales which mirror several groups of psychotic symptoms: Unusual Experiences (UnEx; positive symptoms), Cognitive Disorganization (CogDis; cognitive symptoms), Introvertive Anhedonia (IntAn; negative symptoms), and Impulsive Nonconformity (ImpNon; impulsive and antisocial symptoms). For the purpose of evaluating the suitability of schizotypy as an endophenotype of schizophrenia the current version of the O-LIFE was translated into German: its psychometric properties (including re-test reliability and construct validity) were examined in a large sample (*n* > 1200) and compared to those of the English original. The German version was both highly reliable and consistent with the original. The study aimed to show that schizotypy as measured by the O-LIFE can indeed be regarded as an endophenotype of schizophrenia in terms of genetic associations regarding relevant dopamine-related candidate polymorphisms of schizotypy [i.e., Val^158^Met-polymorphism of the *COMT* gene, uVNTR of the *MAOA* gene, Taq1A-polymorphism of the *DRD2* gene, VNTR of the *SLC6A3* (*DAT*) gene]. We also wanted to compare the genetic associations of the O-LIFE to those published using other operationalizations of schizotypy. Our results show a large number of significant associations and borderline-significant trends between the O-LIFE sub-scales and a range of genes, thereby supporting using the O-LIFE in the search for endophenotypic markers.

## Introduction

According to the World Health Organization, clinical schizophrenia belongs to the most severe disability class (VII), alongside severe depression, severe migraine, quadriplegia, and terminal cancer (WHO, 2008). Ustun (1999) place schizophrenia third in their rank of disabling effects of health conditions by severity behind quadriplegia and dementia. The disorder causes 8.3/8.0 million years lost to disease (YLD), making up for 2.8/2.6% of total YLD with values for males and females respectively (WHO, 2008). First-episode patients are shown to already present with signs of neurodegeneration and loss of brain connectivity which both correlate with the severity of positive symptoms (Suzuki et al., 2005; Lui et al., 2009). Our own research into the mechanisms of neurodegeneration in schizophrenia suggests that the primary pathogenic process underlying the disorder may well be in effect long before the actual onset of psychotic symptoms (Grant, 2011). This is in line both with the current dopamine hypothesis of schizophrenia (Howes and Kapur, 2009) as well as with findings of significant cognitive deficits which predate the first florid psychotic episode (Addington et al., 2003; Van Os and Kapur, 2009). The premorbid IQ of schizophrenic patients, even years before the onset of psychosis, is estimated on average at one-half of a standard deviation below that of healthy controls (Woodberry et al., 2008). Since cognitive deficits are widely accepted to be more enduring than psychotic symptoms (Vinogradov, 2003; Mueser and McGurk, 2004; Van Os and Kapur, 2009) and are considered a better predictor for clinical outcome than response to treatment, it would seem that a possibility for detection of schizophrenic patients before the onset of actual psychosis could become possible in the near future. On such approach could be the investigation of so-called risk alleles of genes thought to be involved in the gene X environment-interaction of the ethiopathogenesis of schizophrenia (Allen et al., 2008; Van Os and Kapur, 2009; Van Os et al., 2010). Both cognitive deficits as well as the presence of such risk alleles are, however, not specific to schizophrenia and are therefore not solely suitable as predictive criteria. It is therefore necessary to search for valid endophenotypes of schizophrenia, which may predict the individual risk of a person to develop psychotic symptoms over the course of time. Endophenotypes are defined as subclinical state-independent characteristics that have a genetic basis and are in concurrence with the biological basis of the actual disorder (Gottesman and Gould, 2003). Such endophenotypes of schizophrenia exist, for example, in deficits in latent inhibition or prepulse inhibition, but the assessment of these as well as genetic testing as a means of screening for endophenotypes are costly and require laboratory work or specialized experimental apparatuses, wherefore they cannot be considered as efficient means within the health care system. The concept of schizotypy according to the original definitions by Rado (1953) or Meehl (1962) meets the criteria for an endophenotype as laid down by Gottesman and Gould (2003).

We therefore examined whether schizotypy/psychosis proneness, as measured by self-report using the “Oxford-Liverpool Inventory of Feelings and Experiences” (O-LIFE) (Mason et al., 1995; Mason and Claridge, 2006), can also be viewed upon as an endophenotype of schizophrenia. Claridge's concept of schizotypy differs from other conceptualizations in that its items are mainly personality-based and not specifically developed on the background of symptoms of clinical schizotypal (personality) disorder or schizophrenia, as are the schizotypy-inventories of Rust [1988; Rust Inventory of Schizyotypal Cognitions (RISC), limited to “positive schizotypy”] and Raine [1991; Schizotypal Personality Questionnaire (SPQ)]. Relatedly, the O-LIFE is based on a fully-dimensional approach to schizotypy, which contrasts with taxonic/quasi-dimensional models as proposed by Rado (1953) or Meehl (1962). The fully dimensional approach by Claridge suggests an intra-individually stable array of traits, whereby high schizotypy-values increase the risk of developing psychosis and persons with schizophrenia-spectrum disorders show particularly high scores in schizotypy with no clear cut-off or distribution break indicating membership of a risk group. The four subscales of the O-LIFE (Mason and Claridge, 2006) also mirror the groups of symptoms found in psychosis: Unusual Experiences (UnEx; positive symptoms), Cognitive Disorganization (CogDis; cognitive symptoms), Introvertive Anhedonia (IntAn; negative symptoms), and Impulsive Nonconformity (ImpNon; impulsive and antisocial symptoms). The inclusion of the last scale is often questioned, but relates on the one hand to the possible concept of “Einheitspsychose” (Claridge, 1997) and on the other to the often reported increased number of violent offences found in psychotic patients (for a review and meta-analysis, see Large and Nielssen, 2011).

The genetic associations of schizotypy have not been extensively studied and published papers often find negative or ambiguous results. Candidate genes of relevance to dopaminergic neurotransmission with risk-alleles that are also considered in the ethiopathogenesis of schizophrenia that have also been associated with schizotypy are primarily the Val^158^Met-polymorphism (rs4680; Lachman et al., 1996) of the *COMT*-gene (encoding for the catecholamine-degrading enzyme catechol-*O*-methyltransferase, C*O*MT) and the variable number of tandem repeats (VNTR)-polymorphism (Vandenbergh et al., 1992) of the *SLC6A3*-gene (encodes for the dopamine active transporter, DAT). Studies operationalizing schizotypy using the SPQ find higher scores for val/val-homozygotes (rs4680) using healthy male participants (Avramopoulos et al., 2002; Smyrnis et al., 2007) or for samples consisting of, i.a., bipolar patients and first-degree relatives of schizophrenic patients (Schurhoff et al., 2007), whereas others report highest scores in met/met-homozygotes (rs4680) (Sheldrick et al., 2008) and again others find only weak but non-significant effects of either the rs4680 or the rs6265 in healthy participants (Ma et al., 2007), whereby these results usually only refer to certain subscales of the SPQ. A study performed by Ettinger and co-workers (2006) on a small (*n* = 31) sample of Caucasian males using the RISC found non-significantly higher scores in met/met- (rs4680) and 10/10-homozygotes (*SLC6A3*-VNTR). No studies exist to date examining the genetic associations of these or other dopamine-related polymorphisms and the O-LIFE.

There are several interpretations of the ambiguity or lack of results concerning the genetic associations of schizotypy, especially regarding the *COMT* Val^158^Met-polymorphism (rs4680): The val-allele is reported to coincide with a higher activity of the expressed enzyme C*O*MT (Lachman et al., 1996), whereby val/val-homozygotes will have the highest and met/met-homozygotes the lowest rate of catecholamine-degradation through C*O*MT. Since dopamine is, however, degraded in a two-step reaction through C*O*MT and an enzyme tandem consisting of monoamine oxidase (MAO; both isozymes) and aldehyde dehydrogenase (AD) (for a concise description of dopamine-synthesis and degradation see Grant, 2011), high levels of C*O*MT-activity will not lead to complete degradation of dopamine, but rather to the formation of the toxic metabolite 3-methoxytyramine (3-MT), and low levels of C*O*MT-activity will lead to a relative over-activity of MAOs/AD and thereby to the formation of the equally toxic metabolite 3,4-dihydroxyphenyl-acteic acid (DOPAC). It could therefore be argued that both findings of higher schizotypy in val/val- as well as met/met-homozygotes could be valid on the background of dopamine-neurotoxicity in schizophrenia-spectrum ethiopathogenesis (Smythies, 1999, 1997; Grant, 2011). The rate of neurotoxicity would therefore be moderated through the relative activity of MAOs. Since a polymorphism with functional consequences for the activity of MAO-A has been described (*MAOA*-uVNTR, Sabol et al., 1998; Deckert et al., 1999), the effects of the rs4680 may be masked, if the individual genotype of the *MAOA*-uVNTR is not taken into consideration. Also, it could be possible that there is actually a heterosis-effect regarding the rs4680, whereby both val/val- and met/met-homozygotes have a higher risk of psychosis proneness compared to val/met-heterozygotes.

Finally, we assume that the fully dimensional model of schizotypy is inherently better suited for endophenotype-research compared to taxonic/quasi-dimensional models, as is leads to more variance in the population compared to those measures with a “quasi-clinical” background containing more items that may be endorsed by fewer individuals. Additionally, due to findings of high heritability of the O-LIFE-scales (Linney et al., 2003), we expect clearer associations to genetic variations with this inventory, especially with the short scales, that have been generated partly on the basis of item-heritability (Mason et al., 2005).

We therefore translated the O-LIFE into German and attempted to assess its suitability for genetic association studies by examining the effects of the aforementioned dopamine-relevant polymorphisms that have previously been related in literature to either the RISC or the SPQ. We additionally examined the association with the *MAOA*-uVNTR and the *DRD2* Taq1A-polymorphism (rs1800497, Pohjalainen et al., 1998), since these polymorphisms have also repeatedly been shown to have significant influences on dopaminergic neurotransmission.

## Materials and methods

### Sample

The main sample for the test-theoretical analysis of the German version of the O-LIFE was acquired via an email-invitation sent to all members (students, fellows, and administrative/technical employees) of Justus-Liebig-University (JLU), Giessen (Germany), through oral invitations during lectures by Phillip Grant at JLU and THM (Technische Hochschule Mittelhessen, University of Applied Sciences) as well as from a German grammar school (Erftgymnasium Bergheim, North Rhine-Westphalia) through personal contacts of Phillip Grant. The email/personal invitations contained a link to an online-version of the inventory programmed by the authors using the platform soscisurvey.de. This online-version consisted of the German O-LIFE and several screening questions regarding somatic and psychological health, drug use (with special regard to alcohol and nicotine) and medication status. The main sample consisted of 1228 participants (341 male, 887 female) with age ranging from 17 to 75 years (*M* = 27.1, *SD* = 9.47, *MD* = 24).

The sample for the re-test of the O-LIFE was acquired 3 months later in the same fashion as the main sample, whereby in this case all other questionnaires and items except the O-LIFE were omitted in order to reduce the time necessary for participants to answer the items and thereby increase compliance. The re-test sample contained 245 participants (45 male, 200 female) with an age range from 17 to 58 years (*M* = 25.83, *SD* = 8.6, *MD* = 23).

The sample for genetic associations was acquired through the Giessen Gene Brain Behaviour Project (GGBBP) of the Department of Personality Research and Individual Differences at JLU. The GGBBP contains ca. 1800 datasets of participants including various personality inventories and data on several polymorphisms, whereby for legal reasons only those participants were contacted who had signed a respective consent form within the last 5 years prior to the date of data-acquisition. Therefore, as well as due to a high rate of unreturned invitations to fill in the O-LIFE, only ca. 290 participants could be acquired from the GGBBP. This sub-sample consisted of 288 participants (91 male, 197 female) with an age range from 18 to 51 years (*M* = 22.9, *SD* = 4, *MD* = 22).

All genetic and molecular-biological research was approved by the local ethics committee of the psychological faculty at JLU.

### German version of the Oxford-Liverpool inventory of feelings and experiences (O-LIFE)

The inventory was translated into German by Phillip Grant, a bilingual native-speaker of German and English, and retranslated into English by the native-German co-authors. Most items were considered to be adequately translated and the remaining items were modified in order to meet optimal retranslation criteria.

The full version of the O-LIFE contains 104 items loading on four scales: UnEx, IntAn, CogDis, and ImpNon. For the properties of the original English version see Mason and Claridge (2006). The short scales (Mason et al., 2005) are drawn from the full inventory.

### Genotyping

DNA was extracted from buccal epithelia using a standard commercial extraction kit (High Pure PCR Template Preparation Kit; Roche, Mannheim, Germany) in a MagNA Pure LC System (Roche, Mannheim, Germany) in line with participants' entry into the database of the GGBBP.

Genotyping was performed by means of polymerase chain reaction amplification according to standard protocols for the following polymorphisms: Val^158^Met-polymorphisms (rs4680) of the *COMT* gene (encoding for C*O*MT) (Reuter and Hennig, 2005) and *DRD2* Taq1A-polymorphism (rs1800497) of the *DRD2* gene (encodes for dopamine receptor D_2_) (Kirsch et al., 2006). For the *MAOA*-uVNTR-polymorphism genotyping was performed using a fluorescently labeled 5′-primer [adapted from Sabol et al. (1998)] and subsequent capillary-electrophoresis on an ABI 310 System (Applied Biosystems, Germany).

Since *MAOA* is an X-chromosomal gene it also has to be noted that men are generally hemizygous, since they only carry a single X-chromosome. In women, heterozygosity is also functionally difficult to interpret, since it cannot be ascertained which individual X-chromosome is inactivated to a Barr-body in each individual neuron. Heterozygous female participants were therefore also excluded from further functional analyses related to the *MAOA*-uVNTR. Due to the absence of methodological points of critique (personal communication from cell-culture expert Dr. Barbara Ahlemeyer, JLU) regarding the functionality-assessments of the *MAOA*-uVNTR-alleles in the study of Deckert et al. (1999), we chose to follow their functional classification of the 5-repeat allele as highly active regarding gene-expression.

In case of the *DAT* 3′UTR-VNTR-polymorphism, which usually consists of 9- or 10-repeat alleles, those participants with other numbers of repeats were omitted, since these alleles are extremely rare (Vandenbergh et al., 1992). Genotyping was performed using primers adapted from Vandenbergh: forward: 5′-TGTGGTGTAGGGAACGGCCTGAG-3′ and reverse 5′-CTTCCTGGAGGTCACGGCTCAAGG-3′ (TIB MOLBIOL, Germany). The 5′-primer was fluorescently labeled, and amplification was followed by capillary-electrophoresis on an ABI 310 System (Applied Biosystems, Germany).

## Results

### Properties of the German Oxford-Liverpool inventory of feelings and experiences (O-LIFE)

The final sample for the German O-LIFE consisted of 1228 participants (341 male, 887 female). The relative imbalance between the sexes can be ascribed to the high prevalence of women in the student-body at JLU, especially within the psychological faculty. The mean age of the participants was 27.1 years with a standard deviation of 9.47, ranging from 17 to 75 years.

Of these 1228 persons, 245 followed the invitation to participate in a re-test 3 months after the initial invitation. These 245 (45 male, 200 female) had an average age of 25.83 years (*SD* = 8.6), ranging from 17 to 58 years.

The statistical values for the O-LIFE sub-scales are shown in Table 1, including the re-test-reliability coefficients. Although the authors of the O-LIFE suggest not to evaluate or overly interpret the total O-LIFE-score (Mason and Claridge, 2006), we calculated this score for reasons of evaluating re-test-reliability of the whole inventory. It should be noted that this total O-LIFE-score was, however, not used for any further analyses. Due to the fact that this publication is in the English language we chose not to show exemplary items of the German version. Interested parties are welcome to contact the corresponding author in this regard. For exemplary items of the O-LIFE in English see Mason and Claridge (2006) and Mason et al. (2005) for the short scales.

**Table 1:** **Statistical properties of the German O-LIFE and comparison to the original**.

	***i***	***M* (Engl.)**	***SD* (Engl.)**	**Range**	**Cronbach's α (Engl.)**	**Short scale (α; *M* [*SD*])**	**Re-test-reliability (Engl.)**
Unusual Experiences	30	7.11 (8.82)	5.51 (6.16)	0–30	0.86 (0.89)	0.72 (*i* = 12) (2.81 [2.26])	0.84 (>0.7)
Cognitive Disorganization	24	10.09 (10.73)	5.98 (5.87)	0–24	0.88 (0.87)	0.78 (*i* = 11) (4.31 [2.87])	0.85 (>0.7)
Introvertive Anhedonia	27	5.83 (6.38)	4.41 (4.49)	0–25	0.81 (0.82)	0.55 (*i* = 10) (1.51 [1.46])	0.85 (>0.7)
Impulsive Nonconformity	23	7.77 (7.69)	3.54 (4.12)	0–21	0.68 (0.77)	0.57 (*i* = 10) (3.03 [2.07])	0.83 (>0.7)
Total O-LIFE-score	104	31.80	13.46	5–83	/	/	0.89 (>0.7)

Abbreviations i number of variables M arithmetic mean SD standard deviation of M; Engl. respective values of the original O-LIFE; re-test interval was 3 months.

It can be seen that the coefficients of internal consistency (α) and of re-test-reliability are comparable to those of the original version with exception of the scale Impulsive Nonconformity (ImpNon). The mean scores and standard deviations of the scale sum-scores are, however, usually slightly higher than in the British sample.

In order to assess if higher scores, especially in the scale UnEx, resulted from disingenuous answering behavior of some of the participants, we compared the consistency indices of different datasets and subsamples. In all of these analyses α-values were similar and acceptable (data not shown here).

As O-LIFE values are reported to be sexually dimorphic, we performed *t*-tests by sex to see if similar differences could be measured in the German translation (Table 2).

**Table 2:** **Sex-differences in O-LIFE scale sum-scores**.

	***M* (*SD*) female**	***M* (*SD*) male**	***T* (*df*)**	***p* (two-tailed)**
UnEx	7.39 (5.53)	6.37 (5.39)	−2.917 (1226)	0.004
CogDis	10.59 (6.00)	8.79 (5.73)	−4.778 (1226)	<0.000
IntAn	5.44 (4.26)	6.84 (4.62)	5.011 (1226)	<0.000
ImpNon	7.48 (3.50)	8.50 (3.54)	4.546 (1226)	<0.000

Abbreviations M arithmetic mean SD standard deviation of M df degrees of freedom p conditional probability.

Since values are different for the whole sample, it is not surprising that mean values for the male and female subsamples are also not identical to those estimated by Mason and Claridge (2006). It could, however, be shown that the direction of the sexual dimorphism is in line with the original (Mason et al., 1995).

Although the sub-scales of the O-LIFE represent clearly distinguishable facets of psychosis proneness or schizophrenia-spectrum disorders, which do not necessarily need to manifest themselves to an equal degree in all individuals, the scales do, nonetheless, show significant inter-correlations in both the original and the German versions (Table 3).

**Table 3:** **Inter-correlations of the O-LIFE scales**.

	**UnEx**	**CogDis**	**IntAn**	**ImpNon**
UnEx	1	0.481^**^	0.115^**^	0.376^**^
CogDis	/	1	0.351^**^	0.287^**^
IntAn	/	/	1	0.021
ImpNon	/	/	/	1

** p < 0.01.

We analyzed the effects of ageing on the O-LIFE scores to find weak but mostly significant (*p* < 0.05) negative correlations between all four scales and age, whereby only IntAn correlated positively (Table 4). In the female subsample the scales UnEx and IntAn did, however, not correlate significantly with age. The directions of these correlations mirror those found for the English O-LIFE (Mason and Claridge, 2006).

**Table 4:** **Correlations between age and the O-LIFE scales**.

	**UnEx**	**CogDis**	**IntAn**	**ImpNon**
whole sample	−0.078^**^	−0.164^**^	0.078^**^	−0.146^**^
men (*n* = 339)	−0.109^*^	−0.136^*^	0.163^**^	−0.153^**^
women (*n* = 878)	−0.060	−0.166^**^	0.030	−0.156^**^

* p < 0.05;

** p < 0.01.

### Genotype-distributions

Two hundred and eighty-eight participants that had been genotyped for the GGBBP also followed the invitation to fill in the O-LIFE. Note that for the single polymorphisms the numbers of participants do not always add up to 288. This is mainly due to the fact that some participants had joined the GGBBP before this research and respective genotypings were conducted and DNA-samples were no longer available, even though the participants were still willing to fill in the German O-LIFE online. Also, due to the argumentation regarding the repeat polymorphisms of the *SLC6A3*- and *MAOA*-genes in the methods section, certain participants with extremely rare or functionally not clearly attributable genotypes were omitted from the analyses. Finally, one participant could not be genotyped regarding the *COMT* VAl^158^Met-polymorphism, probably due to an unexpected individual variation within the amplified fragment.

Table 5 shows that all examined genotypes are in Hardy–Weinberg equilibrium (Court, 2005–2008).

**Table 5:** **Genotype distributions and fit to the Hardy–Weinberg principle**.

**Polymorphism**	**Allele 1**	**Allele 2**	**1/1-frequency**	**1/2-frequency**	**2/2-frequency**	**χ^2^**	***p***
*COMT* Val^158^Met	Val (G)	Met (A)	62	127	91	1.93	0.16
*DAT*-VNTR	9	10	13	93	160	0.012	0.91
*MAOA*-uVNTR	hi	lo	54	89	118	/	/
*DRD2* Taq1A	A1 (T)	A2 (C)	10	84	187	0.022	0.88

### Analyses of variance and homogenous subgroups

Due to the rationale mentioned in the introduction we performed multivariate analyses of variance to examine genotype-associations regarding the O-LIFE scales without prior classification of expected “risk-alleles.” In order to assess, if either the genetic principle of dominance or recessivity was relevant for a given polymorphism, we performed Bonferroni-corrected *post-hoc* tests to examine, if heterozygotes could be considered as (proximately) equal to either of the homozygous groups (data not shown here). These respective groups were then contrasted and compared to the remaining group in single ex-post-facto *t*-tests. This procedure was performed for the whole sample as well as individually for the two sexes. Only relevant data are shown here for reasons of conciseness. Group means and standard deviations can be viewed in Table 6.

**Table 6:** **Significant (*p* < 0.05) genetic associations and borderline-significant trends for O-LIFE-scales**.

**Polymorphism**	**Scale**	**Group 1 (*n*)**	**Group 2 (*n*)**	***M* (*SD*) group 1**	***M* (*SD*) group 2**	***T* (*df*)**	***p* (two-tailed)**
*COMT*	UnEx	val/val (62)	met + (218)	7.66 (5.27)	6.17 (4.95)	2.06 (278)	0.041
Val^158^Met	UnEx sh			3.50 (2.33)	2.64 (2.24)	2.64 (278)	0.009
*COMT*	UnEx sh	val/val male (20)	met + male (65)	3.55 (2.24)	2.45 (2.19)	1.96 (83)	0.053
Val^158^Met							
*COMT*	UnEx sh	val/val female (42)	met + fem. (153)	3.48 (2.40)	2.73 (2.26)	1.88 (193)	0.061
Val^158^Met							
*DRD2* Taq1A	ImpNon	A1/A1 male (4)	A2 + male (81)	6.00 (1.41)	9.10 (3.71)	−3.8 (5.37)	0.011
*MAOA-*uVNTR	CogDis	low functional (54)	high functional (118)	10.59 (6.21)	8.93 (5.84)	1.7 (170)	0.091
*MAOA*-uVNTR	CogDis	low functional	high functional	11.36 (6.72)	8.35 (5.66)	2.03 (80)	0.046
	IntAn	male (22)	male (60)	7.45 (4.63)	4.70 (3.36)	2.6 (29.5)	0.016
	IntAn sh			2.36 (1.73)	1.52 (1.32)	2.36 (80)	0.021
*DAT* 3′UTR-VNTR	UnEx	9/9 (13)	10 + (253)	4.85 (2.67)	6.57 (5.11)	−2.1 (19.9)	0.048
*DAT* 3′UTR-VNTR	UnEx	9/9 female (10)	10 + fem. (176)	4.70 (2.71)	6.72 (5.10)	−2.15 (12.95)	0.051
*DAT* 3′UTR-VNTR	CogDis	9/9 male (3)	10 + male (77)	11.67 (0.58)	9.03 (6.27)	3.35 (40.3)	0.002

Abbreviations n sample size M arithmetic mean SD standard deviation of M df degrees of freedom p conditional probability.

### Genetic associations for the *COMT* Val^158^Met-polymorphism

The analyses of variance regarding the effects of the *COMT* Val^158^Met-polymorphism showed associations with both the full (*p* = 0.092) and the short scales (*p* = 0.031) for UnEx in the whole sample.

In ex-post-facto *t*-tests we found a recessive effect of the val-allele in that the val/val-group showed higher values compared to carriers of the met-allele. This effect was significant for the whole sample (full scale: *T*_278_ = 2.057; *p* = 0.041 and short scale: *T*_278_ = 2.639; *p* = 0.009) and could also be seen in the male and female subgroups, whereby only the values for the short scale of UnEx reached borderline-significance (males: *T*_83_ = 1.964; *p* = 0.053 and females: *T*_193_ = 1.881; *p* = 0.061).

In a general linear model-analysis of the UnEx full and short scales with sex entered as a covariate the recessive effect of the val-allele could also be found (full scale: *F*_1_ = 4.337; *p* = 0.038 and short scale: *F*_1_ = 7.027; *p* = 0.008).

### Genetic associations for the *DRD2* Taq1A-polymorphism

Significant effects of the *DRD2* Taq1A-polymorphism could neither be shown for the whole nor the female sample. Within the group of male participants a significant effect of the A1-allele could be found with A1/A1-homozygote males showing significantly lower values in ImpNon (*T*_5.367_ = −3.785; *p* = 0.011). Due to the fact that this group, however, only consisted of four individuals, this effect shall not be interpreted further.

### Genetic associations for the *MAOA*-uVNTR-polymorphism

Due to the aforementioned argumentation that heterozygous men do not exist and heterozygous females were excluded from the analyses, since their MAO-A-functionality cannot be clearly ascertained, no analyses of variance were performed for this polymorphism.

Within the whole sample a trend could be seen, whereby the low-functional genotype-group showed higher values in CogDis compared to the high-functional group (*T*_170_ = 1.697; *p* = 0.091). No such effect was found in the female sample, but the males showed the same effect in a significant fashion (*T*_80_ = 2.030; *p* = 0.046).

Additionally, the male subsample showed significantly higher values in both the full and short IntAn-scales in the low-activity group (full scale: *T*_29.5_ = 2.557; *p* = 0.016 and short scale: *T*_80_ = 2.359; *p* = 0.021).

**Figure 1:**
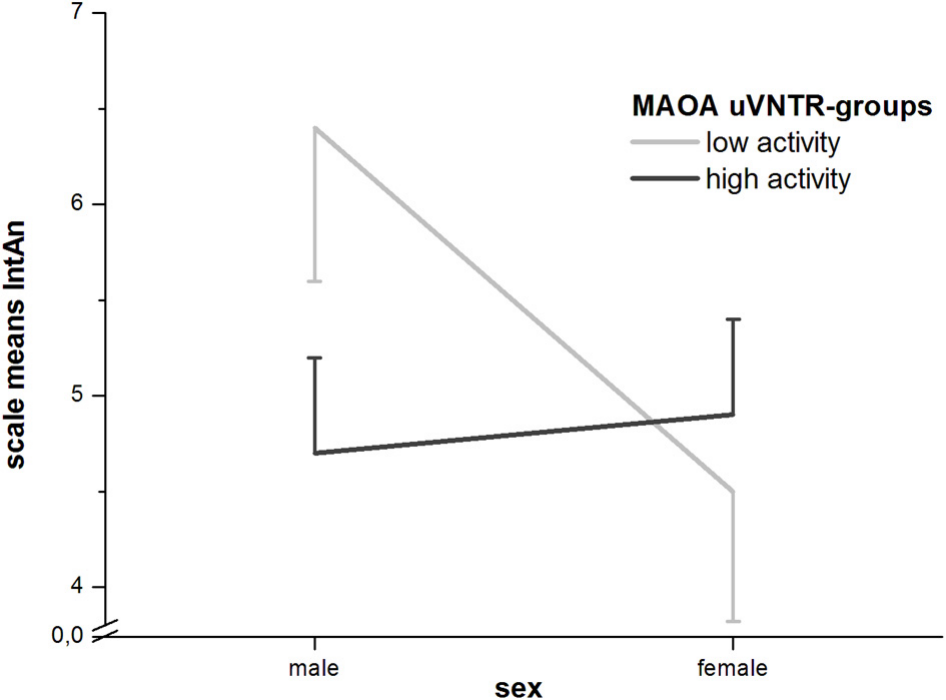
**Sex by MAOA-uVNTR-interaction for IntAn (with standard errors of the means)**.

In GLM-analyses of the whole sample with sex as a covariate there were still no significant effects of the *MAOA*-uVNTR. When sex was, however, entered as a second factor, a significant interaction with the polymorphism could be found for the full and short IntAn-scales (full scale: *F*_1_ = 5.91; *p* = 0.016 and short scale: *F*_1_ = 3.890; *p* = 0.05). Where males with a low-activity genotype showed higher IntAn-scores, females showed equal or even lower (for the full scale) scores in the low-activity group (see Figure 1).

### Genetic associations for the *DAT* 3′UTR-VNTR-polymorphism

Analyses of the main genotype-groups of the *DAT*-VNTR found in our sample (9/9- and 10/10-homozygotes as well as 9/10-heterozygotes) indicated that the relatively rare 9-repeat-allele could have a recessively protective effect on UnEx. Due to this allele's rarity, however, the whole sample only had 13 individuals in this group, 10 of which were female and 3 male. Like in the case of the *DRD2* Taq1A-polymorphism this effect should therefore not be overly interpreted. Lower scores for 9/9-homozygotes were found in the whole sample (*T*_19.91_ = −2.133; *p* = 0.048) and in both sexes, whereby only the female subsample reached borderline-significance (*T*_12.95_ = −2.147; *p* = 0.051). The relatively small group of three 9/9-homozygous males did, however, show an indication that the 9-repeat-allele could be unfavorable regarding the CogDis-scale (*T*_40.27_ = 3.348; *p* = 0.002).

## Discussion

### Preliminary properties of the German O-LIFE

Our first results from a sample of 1228 individuals (341 male, 887 female) show that the German translation of the “O-LIFE” may be considered internally comparable to the original English version. Cronbach's coefficients of internals consistency (α) for the sub-scales UnEx, CogDis, and IntAn are almost equal to those reported by Mason and Claridge (2006). Research on the effects of sample size on Cronbach's alpha suggests that values may rise in samples larger than 130 (Javali et al., 2011). It could therefore be possible that the slight decrease in values of 0.01–0.03 may be attributable to the comparatively larger sample (*n* = 1926) used for the extended norms of the English O-LIFE. The consistency-value for the scale ImpNon, however, is merely 0.68 compared to 0.77 in the English version. A possible explanation herefore may be of a philological and/or psycholinguistic nature: While Old English as well as German were both Germanic languages, Modern English has been influenced massively by both invasions into Britain (notable the Norman Conquest in 1066) as well as through the expansion of the British Empire and the resulting influx of other non-Germanic vocabulary (Shippey, 2000, 2003; Lamb, 2010). Modern English therefore has an extensively larger vocabulary than Modern German, wherefore “finer points” may be slightly lost when using a rather literal approach to item-translation. Several items of the ImpNon-scale consist of words of which the German translations may imply slightly different meanings to different users, as they are not as specific as their Modern English equivalents. For example words like “urge,” “cheat,” “annoy,” “take advantage of,” or “overindulge” were translated as “Drang,” “betrügen,” “ärgern,” “ausnutzen,” and “übertreiben,” respectively. Although these words are quite literal translations, they lack the finer nuances of their Modern English equivalents. The word “overindulge,” for example, does not have a literal equivalent in German and can only be circumscribed or translated as “übertreiben” which in term would literally re-translate as “exaggerate.” It would therefore seem necessary to re-examine specific items (not only from the scale ImpNon) and find less literal but therefore possibly more contentually unambiguous translations. Unfortunately, less literal translation would necessarily mean moving away from the original item.

The average scale sum-scores are also similar to the English original with slightly higher means in UnEx, CogDis, and IntAn. Standard deviations were marginally equal to the original, albeit higher in ImpNon. This is likely to be a sampling effect and would also explain the weaker correlations with age compared to the extended norms of the English O-LIFE (Mason and Claridge, 2006). Our sample consisted mainly of students and is relatively young on average, which explains the higher means for UnEx, IntAn, and CogDis in this case. The fact that CogDis does not increase with age may be explained through considerations that cognitive disorganization is firstly unlikely to be found high in the older participants in our sample, who are mainly fellows of JLU, and secondly due to findings that CogDis is negatively related to creativity (Batey and Furnham, 2008), which is also likely to be higher than average in university-students and probably even more so in university-fellows. Nettle (2006) found that mathematicians show higher scores in IntAn and lower scores in CogDis compared to non-mathematicians. If this is extrapolated for (natural) scientists in general (e.g., psychologists, physicists, statisticians, technicians, etc.), who comprised an over-proportionate part of our sample—especially in the higher-age groups—compared to the average population, the negative correlation for CogDis and positive correlation for IntAn with age is not surprising in our sample. We are currently trying to increase our sample in the context of students' bachelor's theses to include more non-academics as well as more young (<20) and older (>60) individuals before attempting to publish a more valid set of norms for the German O-LIFE.

Apart from these differences, reliabilities (both internal consistencies and test–retest-correlations) as well as inter-correlations between scales are more than acceptable and mirror the findings regarding the original O-LIFE. The reliability and validity results here suggest that the German O-LIFE is a reasonable approximation to the original regarding its capability of measuring schizotypy or “psychosis proneness.” It can therefore be used in our effort to show that the fully dimensional model of schizotypy is well suited as an endophenotype of psychosis or schizophrenia.

### Genetic associations with the O-LIFE sub-scales

We found a number of significant associations and borderline-significant trends regarding individual scales of the O-LIFE for various dopaminergic polymorphisms in the genes *COMT*, *DRD2*, *MAOA*, and *SCL6A3* (*DAT*). We consciously chose not to predict effects of specific “risk”-alleles firstly due to partly ambiguous reports from literature and secondly because we deliberately did not want to exclude the often-overlooked possibility of heterosis-effects, which refer to a general finding that hybrid-species as well as heterozygosity on the genetic level tend to show higher values in advantageous (positive heterosis) and/or lower values in disadvantageous (negative heterosis) traits (for a review on molecular heterosis, see Comings and Macmurray, 2000). Our results did not, however, show significant heterosis-effects, even though we could find possible indications thereof for the rs4680 (see end of following paragraph).

**Figure 2:**
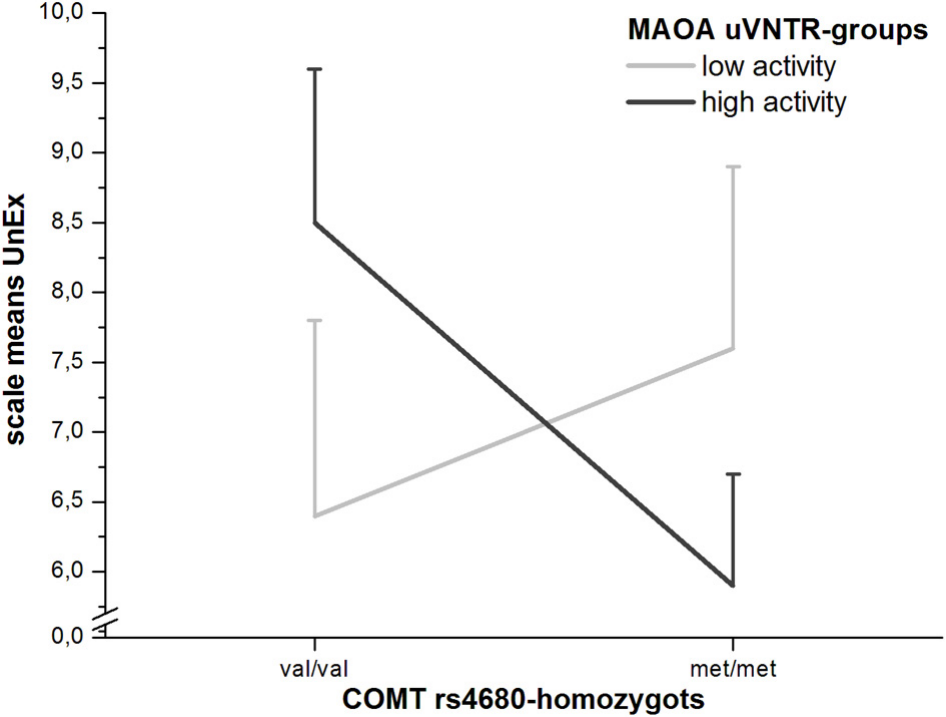
**Moderation of COMT Val^158^Met-effects on UnEx by MAOA-functionality (with standard errors of the means)**.

Higher scores in both UnEx-scales for participants homozygous for the val-allele of the *COMT* Val^158^Met-polymorphism (rs4680) are in line with previous findings (Avramopoulos et al., 2002; Schurhoff et al., 2007; Smyrnis et al., 2007). The val-allele of the rs4680 leads to a higher-active and more thermostable form of the resulting enzyme C*O*MT (Lachman et al., 1996), which is primarily responsible for the degradation of dopamine in the frontal cortex (Karoum et al., 1994). At first, the finding of higher degradation of dopamine leading to higher scores in positive schizotypy may seem contra-intuitive regarding the dopamine-hypothesis of schizophrenia and has also been noted by the authors of the aforementioned papers finding this result. Considering the newest version of the dopamine-hypothesis (Howes and Kapur, 2009), however, this effect does not seem surprising, but actually logically explainable: Under the assumption that schizophrenic and maybe also schizotypal traits do not result from a general over-abundance of dopamine in the frontal cortex and basal forebrain (mainly the accumbens nucleus), but rather from an increased firing rate of the mesolimbic system in the sense of aberrant salience, which in turn is caused by or at least mediated through a reduction in reciprocal prefrontal inhibition of the ventral tegmental area (VTA) (Grant, 2011), pieces start falling into place. Within the prefrontal cortex (PFC) the rate of production of 3-MT, the *O*-methylated metabolite of dopamine, is extremely high, suggesting a disequilibrium of C*O*MT and MAOs (Karoum et al., 1994). Since 3-MT is highly neurotoxic, an increase in its production by the high-active and thermostable variant of C*O*MT, encoded by the val-allele of the *COMT* gene, will lead to increased levels of dopamine-neurotoxicity, atypical frontal neurodegeneration and thereby loss of frontal inhibition of the VTA (Grant, 2011). The question why the effect of val/val-homozygosity is less pronounced in the male and female subgroups and only reached borderline-significance for the short UnEx-scale is harder to explain. Firstly, the O-LIFE short scales were developed based on heritability findings regarding the over 100 O-LIFE items (Linney et al., 2003) and consist of mainly those items with the highest heritability (Mason et al., 2005). It is therefore not surprising that genotypal associations are partly more pronounced in the short compared to the full scales, although this was not the case for all of our findings. Secondly, we also found UnEx-scores to be (non-significantly) lowest in val/met-homozygotes for the whole sample as well as both the male and female subsamples, whereby the sex-difference for UnEx was most pronounced in this group, indicating that men profit more from rs4680-heterosis than women. This effect was increased when the ambiguous group of (female) *MAOA*-heterozygotes was excluded from the analysis. We therefore performed an ex-post-facto GLM-analysis under the exclusion of *MAOA*- and *COMT*-heterozygotes and found a trend-interaction of the two genes on UnEx (*F*_1_ = 2.617; *p* = 0.11) (Figure 2).

Although this effect is not significant, likely due to the sample being too small for analyses of gene by gene interactions, especially under the exclusion of a major number of *MAOA*- and rs4680-heterozygotes, the differences in group means appear to support the role of dopamine-neurotoxicity in the development of (in this case positive) schizophrenic and schizotypal traits and could help explain the findings of Sheldrick et al. (2008) and Ettinger et al. (2006) in whose studies met/met-homozygotes of the rs4680 had the highest schizotypy-scores. The hypothetical model would be that increased C*O*MT-activity (val/val-group) in the frontal cortex might lead to increased dopamine-neurotoxicity and thereby to reduced inhibition and increased firing of the VTA, which in turn would lead to increased presynaptic accumulation of DOPAC (the dopamine-metabolite of MAOs), especially in the high-activity *MAOA*-group. This also leads to an increase in intracellular H_2_O_2_-production [a byproduct of MAO-activity (Maker et al., 1981)], thereby exacerbating dopamine-neurotoxicity and possibly leading to a reduction of presynaptic auto-regulatory mechanisms through the DAT, the vesicular monoamine transporter 2 (VMAT2) and the D_2_-autoceptor (Grant, 2011). The met/met- + high-activity *MAOA*-group would have the lowest levels of toxic dopamine metabolites due to the disequilibrium of C*O*MT and MAOs in the frontal cortex. In the group with high C*O*MT but low MAO-A-activity, presynaptic auto-regulation would likely be better functioning than in the high *MAOA*-group, wherefore schizotypal traits and schizophrenic symptoms would be less pronounced than in the group with high activity in both enzymes but still more pronounced than in the met/met-group of the rs4680 with high MAO-A activity. The last group with low activities in both C*O*MT and MAO-A would have the highest levels of synaptic dopamine and thereby moderately high UnEx-scores due to the abundance of dopamine itself as well as to an increased rate of dopamine-quinone formation (Graham, 1978; Graham et al., 1978), a secondary mechanism in dopamine-neurotoxicity (Grant, 2011). It has to be noted that this model is based solely on neurochemical properties of dopamine-metabolism/-catabolism and has not yet been entirely proven in schizophrenic patients, merely in animal- and *in-vitro*-studies. It does, however, fit to and best explain the findings regarding the genetic associations of *COMT*- and *MAOA*-polymorphisms in this and other studies. Larger studies with sufficient sizes of all relevant groups as well as research using different methods are necessary to examine the verisimilitude of this model further.

Analyses of the singular association of the *MAOA*-uVNTR-polymorphism show a trend toward higher CogDis-values for the low-functional group in the whole sample. The association became significant in the male sample, but not in the female sample, although the direction of this trend was the same in all samples. This effect is explainable due to the enhancing effect of dopamine on cognitive functions and the neurotrophic effect of dopamine during brain ontogeny (for a review, see Nieoullon, 2002). Overall, *MAOA*-genotype had no effects in the female, yet additional effects in the male sample in the full and short IntAn-scales. We therefore examined the possibility of genotype by sex interactions and found these for both scales (full IntAn: *F*_1_ = 5.91; *p* = 0.016 and short IntAn: *F*_1_ = 3.89; *p* = 0.05; q.v. Figure 1). While men with a low-activity *MAOA*-genotype show significantly higher levels than those in the high-activity group for both IntAn-scales, this effect is not found (for the short scale) or even reversed (for the full scale) in women, whereby the differences between genotype-groups was not significant in the female sample. In an animal model, early postnatal MAO-A-inhibition using clorgiline lead to significant reduction in total ambulatory time, rearing behavior and a general increase in neophobia (e.g., in a novelty-suppressed feeding paradigm) compared to vehicle-treated animals. That is, behavioral changes that can be considered upon as similar to an increase in introversion and anhedonia, as well as increased levels of striatal dopamine/DOPAC and decreased levels of serotonin (5-HT) and its primary metabolite 5-hydroxyindoleacetic acid (5-HIIA), whereby these effects were not found, when MAO-A was inhibited in adolescent or adult animals (Yu, 2012). Similar effects on behavior were found by Bortolato et al. (2011) in an incomplete (hypomorphic) knock-out of the *Maoa*-gene in mice (the gene *MAOA*/*Maoa* is highly conserved in many Eutheria, i.e., *Homo sapiens*, *Pan troglodytes*, *Rattus norvegicus*, and *Mus musculus*). These animals were distinctly different from complete *Maoa*-knockouts in that they still showed low, yet detectable enzymatic activity and showed dysphoria- or depression like behavior (e.g., reduced locomotion, grooming, and social interaction) but not higher levels of aggression. Human studies linking the *MAOA*-uVNTR to affective disorders are rare, but a study in healthy female Korean nursing students found a non-significant increase in Beck's Depression Inventory scores when comparing 4/4 repeats (high activity), 3/4 repeats (classified as low activity in this study) and 3/3 repeats (low activity) (Yang et al., 2007). Unfortunately, the classification of the 3/4-group is highly questionable due to the argumentation mentioned in the materials and methods section of this paper. If the resulting means, standard deviations and sample sizes (low activity: *M* = 8.46, *SD* = 6.74, *N* = 79; high activity: *M* = 6.49, *SD* = 6.77, *N* = 43) are used for a one-tailed *t*-test, a *p*-value of 0.063 is found. These results do not, however, explain the interaction with sex in our sample, which is primarily attributable to the high IntAn-scores for low-activity males. We therefore performed exploratory hypothesis-testing on the single item level to find that there was not one item which showed a significant difference in group means for the female sample. In men, however, we found that all items with significant or borderline-significant differences between the high- and low-activity *MAOA*-uVNTR-groups related to social closeness (e.g., making new friends, going out with others, being touched by friends or having intense relationships with others etc.) and behaviors that some men might consider “effeminate” (e.g., enjoying dancing, singing, promenading). It seems therefore that high IntAn-values in males may actually be caused by negation of items referring to social closeness and “unmanly” behavior, which could be in line with findings linking the low-activity variants of the polymorphism in combination with negative life events (in this case possible and severe childhood maltreatment) to higher scores in antisocial personality disorder (Caspi et al., 2002).

A fundamental basis of antisocial personality or psychopathy is the incapability of experiencing fear and learning from errors. The latter has been shown by members of our group to be associated with the A1-allele of the *DRD2* Taq1A-polymorphism (Klein et al., 2007). This allele is linked to reduced density of postsynaptic D_2_-receptors. A study by Hamidovic et al. (2009) found two other SNPs in the *DRD2*-gene as well as their combined diplotype to be associated with high impulsivity and poor behavioral control in those groups with reduced *DRD2*-expression in healthy subjects. The finding that the group of A1/A1-homozygous men had significantly lower results in ImpNon-scores is therefore surprising. Due to the small number of participants in this group (*n* = 4) we chose, however, not to interpret this result further and only report it for the sake of completeness.

Regarding the *SLC6A3* (*DAT*) 3′UTR VNTR-polymorphism, we found lower scores in UnEx in the whole sample and in the female subsample for persons homozygous for the 9-repeat-allele compared to carriers of one or two 10-repeat-alleles. Studies comparing carriers of the 9-repeat-allele to 10/10-homozygotes find weak but non-significant effects regarding lower RISC-scores (Ettinger et al., 2006) and weaker startle magnitudes to various affective stimuli in older adults (Armbruster et al., 2011), which could be indicative of higher affective processing and possible increased salience of affective stimuli in 10/10-homozygotes. Again others report no significant association between the *SLC6A3*-VNTR and schizophrenia (Hauser et al., 2002). In our sample a comparison between 10/10-homozygotes and carriers of one or two 9-repeat-alleles yielded no significant differences. On the other hand, the 9/9-genotype is very rare and was only found in 10 women and 3 men within our sample, wherefore these results are also to be interpreted with caution. While Prata et al. (2009) found a significant interaction between the *SLC6A3*-VNTR and the *COMT* Val^158^Met on brain activity in healthy subjects (*n* = 44) and schizophrenic patients (*n* = 41), independently of diagnosis, we could not find this interaction in our sample. We did find, however, that differences between *COMT* Val^158^Met-genotypes were more pronounced in 10/10- and 9/9-homozygotes compared to heterozygotes, although this observation was not statistically significant.

## Summary and conclusion

Using the German translation of the O-LIFE, which appears to measure the same underlying dimensional trait of schizotypy or psychosis-proneness as the original, we found a large number of significant associations and borderline-significant trends between various dopaminergic and schizophrenia-related genes and the facets of the O-LIFE.

We expected to find generally better associations for the short scales compared to the full scales, due to the fact that these were created partially on the basis of heritability studies. It must be admitted, however, that longer scales (of any trait) probably afford better assessment and reduce error variance. It is possible that alternative item selection and exploratory single-item analyses can identify those individual items with greater relevance for genetic associations.

Most importantly, all genetic associations and trends found between the examined genes and the respective O-LIFE-facets are fully explainable on the basis of neuroanatomical, -chemical, -pathological and -developmental findings. These explanations can also be used interchangeably for schizotypal traits as well as for schizophrenia, wherefore we conclude that the fully dimensional schizotypy-model, as measured with the O-LIFE, is a valid endophenotype of schizophrenia or psychosis-in-schizophrenia.

The limitations of this study are mostly inherent to the genotype- and allele-frequencies of the genes we examined. It is therefore necessary to increase groups with extremely low numbers of individuals (e.g., the *DRD2* Taq1A-group A1/A1) in order to make more generalizable statements. Furthermore, our approach to translate the O-LIFE-items as literally as possible may also have lead to decreases in internal scale-consistencies (especially for the scale ImpNon), wherefore single items might need to be re-examined and slightly altered to better fit the intention of the original. Finally, our sample appears to be selective due to a high prevalence of university-students and -fellows. Whether or not this influences the genetic associations cannot be said at this point, although it would appear improbable, since all effects were clearly explainable and in line with the relevant state of the art. Nonetheless, we are currently increasing our sample to include participants not involved in academia and with a wider age-range, in order to generate genuine norms for the German O-LIFE.

We also realize that not all found effects would survive stringent correction for multiple testing, wherefore the results should be interpreted cautiously. Since, however, each statistical test was performed in independent groups due to our ex-post-facto approach, rather than *post-hoc* comparisons of all individual groups with each other, no group entered into any test twice. Therefore, strictly speaking, it could be argued that no multiple comparisons were reckoned. In any case, it is necessary to assess, if these effects can be replicated in the future in other samples as well as by other researchers.

Our findings add to the growing body of evidence that schizotypy (seen as a set of personality dimensions) and schizophrenia share a common biological basis related to genetic susceptibility or risk as well as shared pathological processes in the sense of dysregulation of dopamine-functioning. We are currently establishing a paradigm to hopefully unequivocally assess aberrant salience and incapability of adequate gating and extinction of irrelevant stimuli, which we want to use on healthy volunteers and schizophrenic patients in combination with genetic analyses, analyses of gene-expression and schizotypy-measurements using the O-LIFE. Our aim hereby is to better understand the pre-clinical development of schizophrenia, the transition from high schizotypy to clinical schizophrenia as well as examine the effects of neurodegenerative processes and their attenuation using respective palliative drugs in the hope of adding to the possibility of hopefully soon being able to detect high schizophrenia-risk in (still) healthy patients and thereby allowing for the possibility of clinical intervention before the first florid episode.

### Conflict of interest statement

The authors declare that the research was conducted in the absence of any commercial or financial relationships that could be construed as a potential conflict of interest.
